# Intracellular and Extracellular Antifreeze Protein Significantly Improves Mammalian Cell Cryopreservation

**DOI:** 10.3390/biom12050669

**Published:** 2022-05-05

**Authors:** Jonathan A. Sreter, Thomas L. Foxall, Krisztina Varga

**Affiliations:** 1 Department of Molecular, Cellular and Biomedical Sciences, University of New Hampshire, Durham, NH 03824, USA; jonathan.sreter@unh.edu; 2 Department of Biological Sciences, University of New Hampshire, Durham, NH 03824, USA; tom.foxall@unh.edu

**Keywords:** antifreeze protein, cryopreservation, cryoprotectants, freezing, mammalian cells

## Abstract

Cell cryopreservation is an essential part of the biotechnology, food, and health care industries. There is a need to develop more effective, less toxic cryoprotective agents (CPAs) and methods, especially for mammalian cells. We investigated the impact of an insect antifreeze protein from *Anatolica polita* (ApAFP752) on mammalian cell cryopreservation using the human embryonic kidney cell line HEK 293T. An enhanced green fluorescent protein (EGFP)-tagged antifreeze protein, EGFP–ApAFP752, was transfected into the cells and the GFP was used to determine the efficiency of transfection. AFP was assessed for its cryoprotective effects intra- and extracellularly and both simultaneously at different concentrations with and without dimethyl sulfoxide (DMSO) at different concentrations. Comparisons were made to DMSO or medium alone. Cells were cryopreserved at −196 °C for ≥4 weeks. Upon thawing, cellular viability was determined using trypan blue, cellular damage was assessed by lactate dehydrogenase (LDH) assay, and cellular metabolism was measured using a metabolic activity assay (MTS). The use of this AFP significantly improved cryopreserved cell survival when used with DMSO intracellularly. Extracellular AFP also significantly improved cell survival when included in the DMSO freezing medium. Intra- and extracellular AFP used together demonstrated the most significantly increased cryoprotection compared to DMSO alone. These findings present a potential method to improve the viability of cryopreserved mammalian cells.

## 1. Introduction

Cryopreservation of cells has long been necessary in the use of cells in research, for in vitro fertilization, and the increased use of mammalian cells in the production of therapeutics, vaccines, and even food production [1,2,3,4]. Thus, there is a need for cryoprotectants and improved cryopreservation protocols that will enhance the viability of cells stored at low temperatures, and some of these can be found as naturally occurring proteins [5]. Cellular therapies offer precise, potent, and cutting-edge treatment options for complex diseases [6,7,8,9,10,11]. However, reliable and consistent long-term cryogenic storage of mammalian cells remains a challenge, and it has been recognized as a potential major obstacle in the development of complex cellular therapies [12,13]. Long-term storage and transport can also be further complicated, as some cells are especially sensitive to freezing damage [14]. For over 60 years, dimethyl sulfoxide (DMSO) has been added to cells to reduce ice formation when stored in liquid nitrogen (−196 °C); however, DMSO can have harmful effects by causing adverse reactions in patients and exhibiting cellular toxicity [15,16].

Almost all cells and cellular organisms are damaged by freezing, and much is still not understood about how to counteract its adverse effects. Numerous organisms have natural compounds to avoid or tolerate freezing in order to survive in extreme cold. Antifreeze proteins (AFPs), a type of ice-binding protein, were first discovered in Antarctic fish blood [17]. In recent decades, it has been discovered that a wide range of organisms produce AFPs for protection against freezing [18,19,20]. Mammals, however, have not been found to produce AFPs. AFPs inhibit ice growth, direct ice crystal shaping, and prevent ice recrystallization in cold-adapted organisms as a result of becoming adsorbed to the nascent ice surface by an unusual mechanism [21,22,23].

Previous work has investigated incorporating AFPs as cellular cryoprotectants [19,24,25,26,27,28,29]. However, these studies typically involved teleost or other moderately active AFPs that shape ice crystals into needle-like formations that can puncture cell membranes [25,30]. Insect AFPs induce formation of rounded ice crystals, which may reduce cell membrane damage and provide better cryoprotective activity [31]. To date, these insect AFPs have been added to cryoprotective agents (CPAs) as a non-penetrating part of cryoprotectant solutions, and some have shown promising results in mammalian cells [32,33].

The insect antifreeze protein ApAFP752 was found in the central Asian desert beetle *Anatolica polita* [34]. These deserts can experience extreme temperature fluctuations up to +40 °C and down to −40 °C in a day [34,35,36]. ApAFP752 is a 9 kDa protein with a predicted highly disulfide bonded β-helical structure containing an array of Thr residues on the ice-binding surface, similar to the homologous AFP from the beetle *Tenebrio* *molitor* (TmAFP) [37,38]. Current studies of ApAFP752 use the recombinant thioredoxin A (TrxA) fusion protein TrxA-ApAFP752 [24,34,39,40].

TrxA-ApAFP752 has previously been shown to provide cryoprotection to *E. coli* cells against cold damage [34], *Xenopus laevis* eggs [41] and human skin fibroblast cells [24,39,40]. In these studies, TrxA-ApAFP752 demonstrated cryoprotective activity when microinjected into the *Xenopus laevis* eggs [41], and when included in the freezing medium external to the *E. coli* [34] and human skin fibroblast cells [40]. In this study, the goal was to observe and determine both the extracellular and intracellular cryoprotective activity of ApAFP752 in human cells. We designed plasmids for both an enhanced green fluorescent protein (EGFP–ApAFP752) fusion protein and EGFP alone. Cells were then left untransfected or transfected with either EGFP–ApAFP752 fusion protein or EGFP alone and the transfection efficiency and cryoprotective activity were assessed compared to varying concentrations of DMSO. Extracellular Trx-ApAFP752 will be denoted as EC AFP, and transfected EGFP–ApAFP752 will be referred to as intracellular or IC AFP. Extracellular TrxA-ApAFP752 (EC AFP) and intracellular EGFP–ApAFP752 (IC AFP) cryoprotection efficacy was then evaluated and compared using trypan blue for viability determination, lactate dehydrogenase (LDH) release for membrane damage, and metabolic activity from the cellular metabolism of (3-(4,5-dimethylthiazol-2-yl)-5-(3-carboxymethoxyphenyl)-2-(4-sulfophenyl)-2H-tetrazolium (MTS) assays.

## 2. Materials and Methods

### 2.1. Cells and Reagents

Human embryonic kidney (HEK) 293T cells were purchased from American Type Culture Collection (ATCC, Manassas, VA, USA), and cultured in Dulbecco’s Modified Eagle Medium (DMEM) (Thermo Fisher Scientific, Waltham, MA, USA) supplemented with 10% *v*/*v* fetal bovine serum (Sigma-Aldrich, St. Louis, MO, USA). Cells were incubated in a humidified incubator at 37 °C and 5% CO_2_. Cells were rinsed with Dulbecco’s phosphate-buffered saline (DPBS) (Thermo Fisher Scientific, Waltham, MA, USA) and dissociated from cultureware using 0.05% trypsin + 0.02% EDTA (Thermo Fisher Scientific, Waltham, MA, USA). Cell viability was determined using a hemocytometer and 0.4% trypan blue solution (Thermo Fisher Scientific, Waltham, MA, USA) and an Invitrogen Countess™ II FL automated cell counter (Thermo Fisher Scientific, Waltham, MA, USA).

### 2.2. Transfection of HEK 293T Cells

Plasmids for EGFP and EGFP–ApAFP752 were designed and purchased from GeneArt (Thermo Fisher Scientific, Waltham, MA, USA). Transfection of EGFP–ApAFP752 plasmid (Figure S1) into HEK 293T cells was optimized according to manufacturer protocols. After optimization, 1 × 10^7^ cells were seeded into T75 flasks with a final volume of 19.7 mL DMEM supplemented with 10% FBS and incubated overnight. The following day, cells were ~80% confluent. A volume of 20 µL of plasmid DNA (1 µg/µL) was combined with 2 mL of Gibco™ OptiMEM (Thermo Fisher Scientific, Waltham, MA, USA), then 60 µL of TransIT^®^-293 (Mirus Bio, Madison, WI, USA) was mixed in and left for 30 min at room temperature to complex. The solution was then mixed, added to the ~80% confluent T75 flask of HEK 293T cells, and cells were incubated for 48 h. Following incubation, cells were observed, and digital images were taken with epifluorescence microscopy to visualize EGFP and EGFP–ApAFP752 transfection.

### 2.3. Flow Cytometry

After visualization of optimal transfection conditions, transfection efficiency was quantified by measuring EGFP expression using flow cytometry. Transfected HEK 293T cells were released by trypsinization, centrifuged, and resuspended in DPBS. Flow cytometry was performed using a Sony SH-800Z sorting flow cytometer (Sony Biotechnology, San Jose, CA, USA) equipped with 405, 488, 561, and 638 nm lasers capable of detecting up to 8 parameters (6 fluorescent and 2 scatter channels). Sony cell sorter software was used to operate the instrument. Untransfected HEK 293T cells were used as a negative control. The 488 nm excitation laser was used with a 100 µM chip operating at 6 psi. EGFP (or EGFP–ApAFP752) fluorescence was detected using the fluorescence 2 (FL2) emission detector at 525 nm. The percentage of HEK 293T cells expressing EGFP (or EGFP–ApAFP752) was measured by gating for a region with <1% of untransfected HEK 293T cells in the EGFP gated region, as described previously [42]. The gating strategy can be found in Figure S2. All flow cytometry data were analyzed using FlowJo software (Becton Dickinson, Franklin Lakes, NJ, USA).

### 2.4. Expression and Purification of Recombinant TrxA-ApAFP752

TrxA-ApAFP752 was expressed and purified for testing it as an extracellular agent (EC AFP) and for comparison with intracellular EGFP–AFP (IC AFP) to determine which has more potent activity in HEK 293T cryopreservation. Expression and purification of TrxA-ApAFP752 were performed as described previously [40]. In short, the recombinant plasmid pET32b-TrxA-ApAFP752 was transformed into BL21 (DE3) pLysS competent *Escherichia coli* cells (Promega, Madison, WI, USA). Protein overexpression was induced with isopropanol-1-thio-β-D-galactopyranoside (IPTG) [34]. The cells were harvested via centrifugation and then lysed using a French press. TrxA-ApAFP752 was purified using an ÄKTA purifier 900 fast protein liquid chromatography (FPLC) system with nickel-affinity columns (Cytiva, Marlborough, MA, USA). Protein purity was assessed using SDS-PAGE and Coomassie blue staining. TrxA-ApAFP752 concentration was estimated using UV-Visible spectrophotometry (ε_280_ = 19,575 M^−1^·cm^−1^). TrxA-ApAFP752 was used as extracellular AFP (EC AFP) at final concentrations of 5 or 15 µM (0.13 or 0.40 mg/mL) in the cryoprotectant solutions.

### 2.5. Cryopreservation and Thawing

Prior to cryopreservation, all HEK 293T cell viability was >90%. For studies comparing untransfected HEK 293T cells, those transfected with EGFP, or those transfected with EGFP–ApAFP752 (AFP), 1 mL of 5 × 10^6^ cells was cryopreserved with 0, 5, 10, 15, and 20% (*v*/*v*) concentrations of DMSO in Corning^®^ cryogenic tubes (Corning, Corning, NY, USA). Cryotubes were placed in a Mr. Frosty™ freezing container (Thermo Fisher Scientific, Waltham, MA, USA) and cooled at −1 °C/min to −80 °C. After 24 h, cryotubes were then stored in liquid nitrogen vapor phase (196 °C) for ≥4 weeks. Cells were rapidly thawed using a 37 °C water bath, added to 5 mL of prewarmed DMEM supplemented with 10% FBS, and centrifuged for 5 min at 200× *g*. The resulting cell pellet was then resuspended in 5 mL of prewarmed DMEM supplemented with 10% FBS.

The cryopreservation and thawing methods described previously were then used to compare the cryoprotective activity of extracellular AFP (EC AFP) and intracellular AFP (IC AFP) and both together. The following cryopreservation conditions were compared to untransfected HEK 293T cells frozen with the same DMSO concentrations (0, 5, and 10% *v*/*v*) and stored in liquid nitrogen vapor phase (−196 °C) for ≥4 weeks:

(1) 5 µM EC AFP,
(2) 15 µM EC AFP,
(3) IC AFP,
(4) 5 µM EC AFP and IC AFP, and
(5) 15 µM EC AFP and IC AFP.

### 2.6. Viability Tests

Three different assays were utilized to assess the cryopreservation efficacy of the HEK 293T cells after freeze/thaw: trypan blue, LDH release, and MTS assays. It is important to note that immediate post-thaw viability testing can fail to account for cellular apoptosis or necrosis in some cells, which may take 24–48 h to occur [43,44]. To increase confidence in results from immediate post-thaw viability assays, such as trypan blue, additional viability assessments were conducted at multiple time points including both immediate- (within 12 h) and longer-term (48 h post-thaw) testing. The trypan blue viability assay is based on the principle that the vital dye, trypan blue, enters dead or dying cells with a damaged membrane while leaving viable cells with intact membranes unstained [45]. Any user error was mitigated by the use of an automated cell counter in conjunction with manual counting with a hemocytometer [46,47]. The LDH release assay is based on the fact that cells undergoing necrosis, apoptosis, or other cellular membrane damage will rapidly release LDH into the surrounding medium, and this is easily quantified by the LDH release assay [48]. Cellular metabolic activity was measured by the reduction of a tetrazolium compound, MTS (3-(4,5-dimethylthiazol-2-yl)-5-(3-carboxymethoxyphenyl)-2-(4-sulfophenyl)-2H-tetrazolium, and an electron coupling reagent (phenazine ethosulfate; PES) by metabolically active living cells to form a colored formazan product [49]. This assay is widely used to determine either cellular proliferation or cytotoxicity by quantifying cellular metabolism. The same number of viable cells counted using trypan blue were plated for each treatment 48 h prior to measurement.

#### 2.6.1. Trypan Blue Assay

All HEK 293T cells were enumerated and viability was determined using a hemocytometer and 0.4% trypan blue vital dye solution (Thermo Fisher Scientific, Waltham, MA, USA) as well as an Invitrogen Countess™ II FL automated cell counter (Thermo Fisher Scientific, Waltham, MA, USA) [45]. Cell viability assessment using the trypan blue assay began within 1 h after freeze/thaw and was completed within 12 h.

#### 2.6.2. LDH Assay

Within 1 h post-thaw, the Cyquant™ LDH cytotoxicity assay (Thermo Fisher Scientific, Waltham, MA, USA) was performed to determine the amount of cell damage by following manufacturer protocols and absorbance values were read at 490 nm using a spectrophotometric plate reader (BioTek, Winooski, VT, USA) [48]. Total LDH released was determined by measuring Triton X-100 lysed HEK 293T cells as positive controls. All values were media subtracted and cell damage was expressed as % total LDH release.

#### 2.6.3. MTS Assay

Viable cells for each condition were plated in a 96-well plate at a density of 1.5 × 10^4^ cells/well. Cell medium was changed after 24 h and a CellTiter 96^®^ Aqueous One Solution Cell Proliferation Assay kit (Promega, Madison, WI, USA) was used to perform an MTS assay 48 h post-thaw according to manufacturer protocols. Absorbance values were read at 490 nm using a spectrophotometric plate reader (BioTek, Winooski, VT, USA). Percent (%) total metabolic activity was measured relative to fresh, non-cryopreserved HEK 293T cells.

### 2.7. Experimental Design and Statistical Analysis

First, the objective was to determine successful transfection of EGFP–ApAFP752 (IC AFP) and EGFP into HEK 293T cells. Next, we determined the cryoprotective effect of IC AFP compared to the control groups of untransfected cells and EGFP-transfected cells at 0, 5, 15, 15, and 20% *v*/*v* DMSO concentrations using three different cell viability measurements (trypan blue, LDH, and MTS). After establishing that AFP is responsible for the intracellular cryoprotective effect, comparisons were then made to determine the cryoprotective activity of extracellular AFP (EC AFP) and intracellular AFP (IC AFP) and both together at 0, 5, and 10% *v*/*v* DMSO concentrations again using three different cell viability measurements (trypan blue, LDH, and MTS). All experiments contained 3 biological repeats (*n* = 3), with each containing 3 technical repeats. Statistical analyses were performed using GraphPad Prism 9 (GraphPad Software, San Diego, CA, USA).

## 3. Results

### 3.1. Transfection

**Figure 1:**
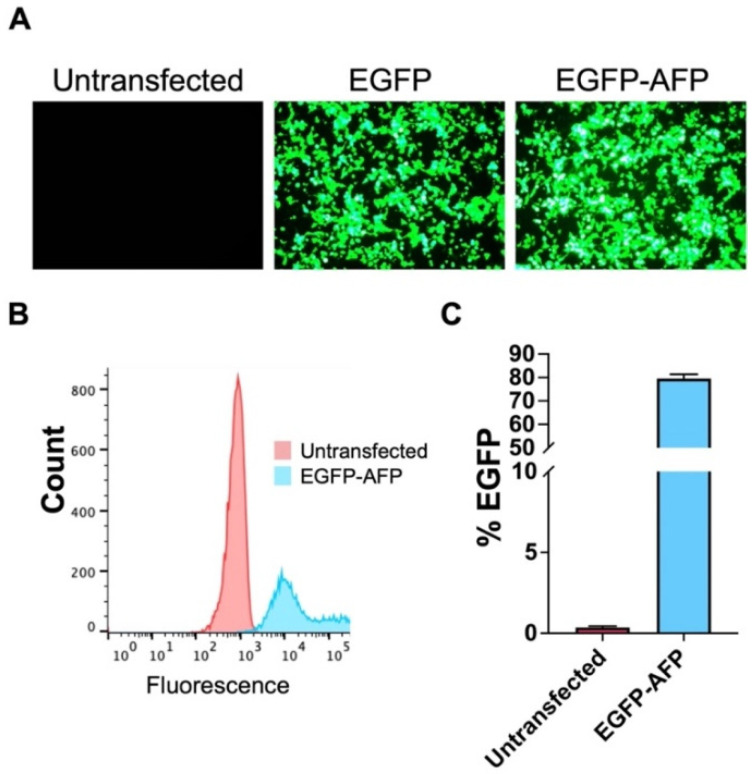
Transfection of HEK 293T cells. (**A**) Epifluorescent images of HEK 293T cells comparing untransfected cells and cells transfected with EGFP and EGFP–ApAFP752 (EGFP–AFP) after 48 h photographed at 100X. (**B**) Flow cytometry analysis of AFP transfection measuring transfection efficiency of EGFP–ApAFP752 gated against untransfected cell autofluorescence. (**C**) Average transfection efficiency (% EGFP expression) for EGFP–ApAFP752 (blue) compared to untransfected cells (magenta). Mean value ± SEM. All experiments contained three biological repeats (*n* = 3), with each containing three technical repeats.

To evaluate transfection efficiency, cells were observed and imaged using both light and epifluorescence microscopy at 24 and 48 h post-transfection (Figure 1 and Figure S3). Cells transfected with EGFP or EGFP–ApAFP752 produced green fluorescence when excited by blue light (450–490 nm). No fluorescence was observed in untransfected cells. The amount of EGFP–ApAFP752 expression was higher after 48 h vs. 24 h, so 48 h was selected for optimal protein expression and absence of cellular pathologies. It should be noted that cells were also examined 72 h post-transfection; however, no increase in protein expression was observed. Flow cytometry was performed to quantify the percent of cells expressing EGFP (or EGFP–ApAFP752) fluorescence (transfection efficiency) and transfection efficiency was determined to be an average of 80%.

### 3.2. Untransfected vs. EGFP vs. IC AFP

**Figure 2:**
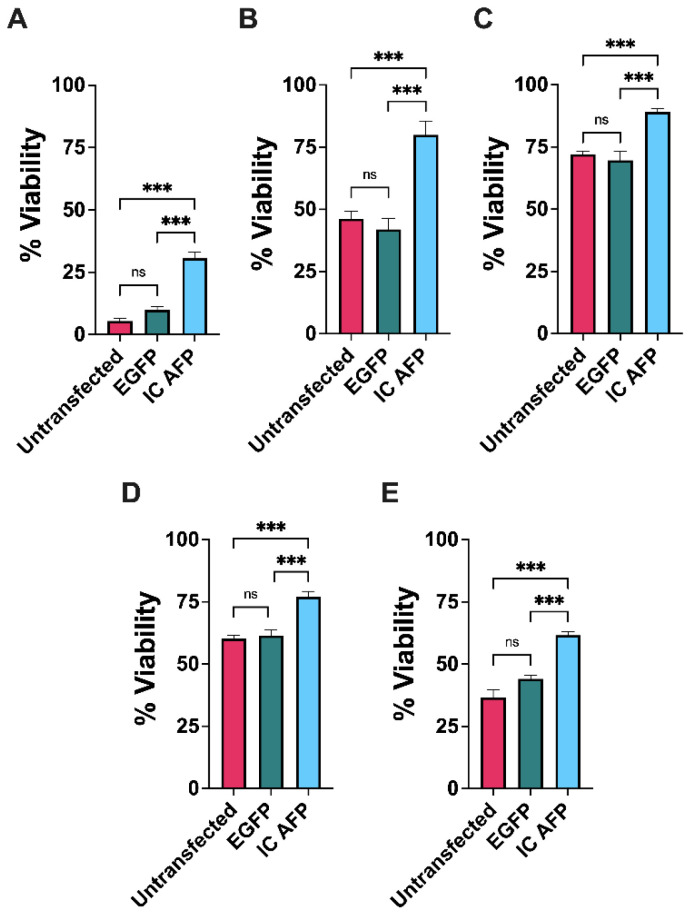
Analysis of cell viability by trypan blue exclusion in untransfected, EGFP-transfected, and EGFP–AFP-transfected (IC AFP) HEK 293T cells after cryopreservation. A total of 5 × 10^6^ cells were cryopreserved with (**A**) 0, (**B**) 5, (**C**) 10, (**D**) 15, and (**E**) 20% (*v*/*v*) concentrations of dimethyl sulfoxide (DMSO) and stored in liquid nitrogen for ≥4 weeks. Data were analyzed using a one-way analysis of variance (ANOVA) and Tukey’s post hoc test was used for pairwise comparisons of experimental groups. All experiments contained 3 biological repeats (*n* = 3), with each containing 3 technical repeats. Mean value ± SEM (n.s. *p* > 0.05, *** *p* ≤ 0.001).

EGFP and untransfected cells are the negative controls to rule out any potential cryoprotective activity from the EGFP part of the EGFP–AFP fusion protein for IC AFP. Untransfected, EGFP-transfected, and EGFP–ApAFP752-transfected cells (IC AFP) were cryopreserved with 0, 5, 10, 15, and 20% (*v*/*v*) concentrations of cell culture-grade DMSO. These concentrations were chosen because 5–10% DMSO are the concentrations most commonly used for cell cryoprotection, and we sought to compare a wide range for determining DMSO activity [50]. Cells were then stored in liquid nitrogen vapor phase (−196 °C) for ≥4 weeks. Cells that were frozen and thawed without cryoprotectants (0% DMSO, untransfected cells), Figure 2A, exhibited lowest viability, ~5% on average (Table 1). The 5% and 10% DMSO increased the survival of untransfected HEK 293T cells; however higher DMSO concentrations of 15% and 20% decreased cell survival, implying the toxicity of higher DMSO concentrations to cells (Figure 2 and Table 1). Post-thaw testing showed a significant increase in viability for cells transfected with AFP (IC AFP) vs. untransfected cells or cells transfected with EGFP across all treatments using the trypan blue viability assay (Figure 2). The results are summarized in Table 1. There were no significant differences between untransfected cells and cells transfected with EGFP across each DMSO concentration.

**Figure 3:**
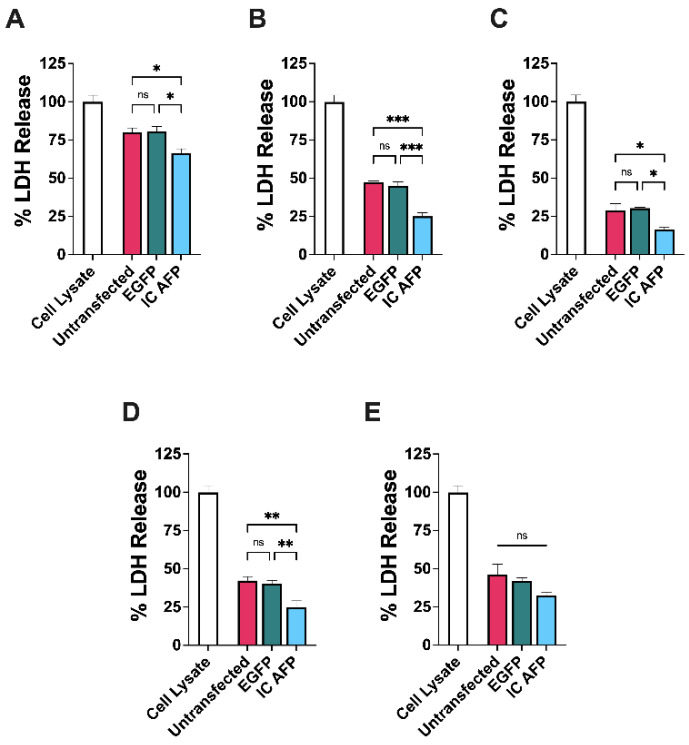
LDH assay of cell damage for untransfected, EGFP-transfected, and EGFP–AFP-transfected (IC AFP) HEK 293T cells after cryopreservation. A total of 5 × 10^6^ cells were cryopreserved with (**A**) 0, (**B**) 5, (**C**) 10, (**D**) 15, and (**E**) 20% (*v*/*v*) concentrations of DMSO and stored in liquid nitrogen vapor phase (−196 °C) for ≥4 weeks. All values are media subtracted and cell damage is expressed as % total cellular LDH. Data were analyzed using a one-way analysis of variance (ANOVA) and Tukey’s post hoc test was used for pairwise comparisons of experimental groups. All experiments contained 3 biological repeats (*n* = 3), with each containing 3 technical repeats. Mean value ± SEM (n.s. *p* > 0.05, * *p* ≤ 0.05, ** *p* ≤ 0.01, *** *p* ≤ 0.001).

Post-thaw cell damage was measured using an LDH assay (Figure 3). The assay showed a significant decrease in LDH release for IC AFP cells vs. untransfected cells, or cells transfected with EGFP across all treatments except 20% DMSO. The findings are summarized in Table 2. There were no significant differences between untransfected cells and cells transfected with EGFP across each DMSO concentration.

**Figure 4:**
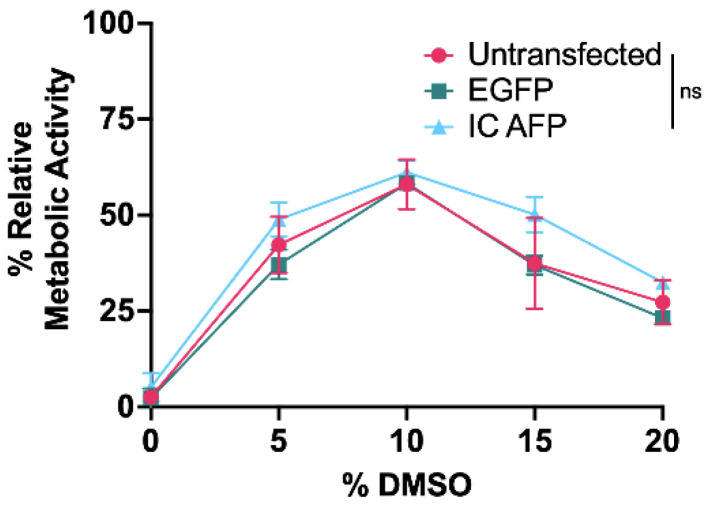
Relative metabolic activity assessed via MTS assay performed on untransfected, EGFP-transfected, and EGFP–AFP-transfected (IC AFP) HEK 293T cells after cryopreservation. A total of 5 × 10^6^ cells were cryopreserved with 0, 5, 10, 15, and 20% (*v*/*v*) concentrations of DMSO and stored in liquid nitrogen vapor phase (−196 °C) for ≥4 weeks. The medium was changed after 24 h and MTS assay was performed after 48 h with % metabolic activity measured relative to fresh, non-cryopreserved HEK 293T cells. Data were analyzed using a one-way analysis of variance (ANOVA) and Tukey’s post hoc test was used for pairwise comparisons of experimental groups. All experiments contained 3 biological repeats (*n* = 3), with each containing 3 technical repeats. Mean value ± SEM (n.s. *p* > 0.05).

The MTS assay showed no significant differences in metabolic activity between untransfected, EGFP-transfected, and AFP-transfected cells for each DMSO concentration. There results indicate both the accuracy of the trypan blue and LDH release assays performed within 12 h post-thaw and the lack of mitogenic effects of AFP for HEK 293T cells (Figure 4).

### 3.3. Intracellular vs. Extracellular AFP

**Figure 5:**
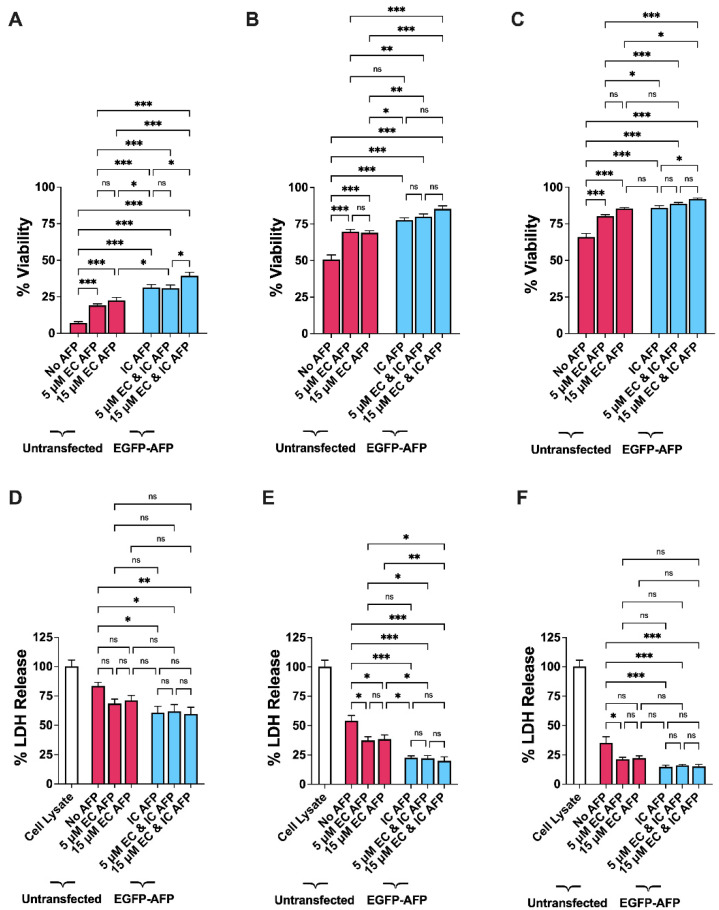
(**A**–**C**) Trypan blue exclusion assay in untransfected (red bar graphs) and AFP-transfected (IC AFP, blue bar graphs) HEK 293T cells after cryopreservation. A total of 5 × 10^6^ cells were cryopreserved with (**A**) 0, (**B**) 5, and (**C**) 10% (*v*/*v*) concentrations of DMSO and/or 5 or 15 µM (0.132 or 0.396 mg/mL) TrxA-ApAFP752 (EC AFP) and stored in liquid nitrogen vapor phase (−196 °C) for ≥4 weeks. (**D**–**F**) LDH assay measurement of cell damage for untransfected (red bar graphs) and AFP-transfected (IC AFP, blue bar graphs) HEK 293T cells after cryopreservation. A total of 5 × 10^6^ cells were cryopreserved with (**D**) 0, (**E**) 5, and (**F**) 10 % (*v*/*v*) concentrations of DMSO and/or 5 or 15 µM (0.132 or 0.396 mg/mL) TrxA-ApAFP752 (EC AFP) and stored in liquid nitrogen vapor phase (−196 °C) for ≥4 weeks. All values are media subtracted and cell damage is expressed as % total cellular LDH in cell lysate (white bar graph). Data were analyzed using a one-way analysis of variance (ANOVA) and Tukey’s post hoc test was used for pairwise comparisons of experimental groups. All experiments contained 3 biological repeats (*n* = 3), with each containing 3 technical repeats. Mean value ± SEM (n.s. *p* > 0.05, * *p* ≤ 0.05, ** *p* ≤ 0.01, *** *p* ≤ 0.001).

In order to determine whether the cryoprotective activity of IC AFP is more potent than extracellular AFP (EC AFP), the two conditions were directly compared to untransfected cells. Due to no increased cryoprotective effects observed for 15 or 20% *v*/*v* DMSO over 5 and 10% *v*/*v* DMSO, and with the aim to use as little DMSO as necessary for cryopreservation, DMSO concentrations were kept at those most commonly used for cryopreservation (5 and 10% *v*/*v*) for intracellular vs. extracellular (IC vs. EC) AFP testing [50]. We tested two concentrations (5 and 15 μM) of EC AFP to establish its efficacy in cryopreservation, and 5 μM was chosen as the minimal concentration as we have previously shown that this was the minimal concentration at which purified TrxA-ApAFP752 exhibited potent ice-recrystallization inhibition (IRI) behavior [41]. The trypan blue assay showed a significant increase in viability for all AFP treatments (Figure 5A–C). Cells cryopreserved with both extracellular and intracellular (EC and IC) AFP compared to untransfected HEK 293T cells yielded the highest levels of cryoprotection. These viability increases are summarized in Table 3.

Post-thaw cell damage was measured using an LDH assay comparing untransfected cells to the various AFP treatments (Figure 5D–F). For 5 µM vs. 15 µM EC AFP, significant decreases in LDH release were not found in 0% DMSO samples (Figure 5D). There was also no significant decrease in LDH release for cells cryopreserved with 15 µM EC AFP at 10% DMSO (Figure 5F). It should be noted that this value is very nearly statistically significant (*p* = 0.06), and this treatment may still be biologically significant. A significant decrease in LDH release was found in all IC AFP treatments and the results are summarized in Table 4 and Table S2.

**Figure 6:**
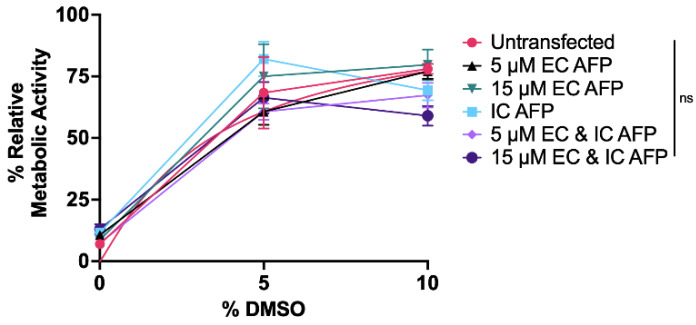
Relative metabolic activity assessed via MTS assay performed on untransfected and AFP-transfected (IC AFP) HEK 293T cells after cryopreservation. A total of 5 × 10^6^ cells were cryopreserved with 0, 5, and 10% (*v*/*v*) concentrations of DMSO and/or 5 or 15 µM (0.132 or 0.396 mg/mL) TrxA-ApAFP752 (EC AFP) and stored in liquid nitrogen vapor phase (−196 °C) for ≥4 weeks. The medium was changed after 24 h and MTS assay was performed after 48 h with % metabolic activity measured relative to fresh, non-cryopreserved HEK 293T cells. Data were analyzed using a one-way analysis of variance (ANOVA) and Tukey’s post hoc test was used for pairwise comparisons of experimental groups. All experiments contained 3 biological repeats (*n* = 3), with each containing 3 technical repeats. Mean value ± SEM (n.s. *p* > 0.05).

The MTS assay showed no significant differences in metabolic activity between post-thaw untransfected cells and EGFP–ApAFP752-transfected (IC AFP) cells for each condition for each DMSO concentration (Figure 6). This indicates the accuracy of the trypan blue and LDH assays performed within 12 h post-thaw and no mitogenic effects of EC AFP or IC AFP for HEK 293T cells.

## 4. Discussion

Effective cryopreservation and long-term storage are essential requirements for the use of cells in research and clinical applications of cell-based therapies, and improving cryopreservation materials and procedures is critical for many cell types [51,52]. There are two major categories of CPAs: penetrating and non-penetrating [50,53]. As their names imply, non-penetrating CPAs are extracellular, with some examples being polymers, such as polyvinyl alcohol (PVA) or polyampholytes [13,54]. Penetrating CPAs such as DMSO or glycerol are intracellular and are the most commonly used of all CPAs [5,55]; and in mammalian cell culture, most cryopreservation procedures utilize DMSO as the cryoprotectant. The concentration of DMSO as well as exposure time must be optimized to a level that yields the most cryoprotective benefit with the least cytotoxic effects, and in most applications, cells are incubated in the presence of 5–10% *v*/*v* of DMSO for 10 min prior to freezing to allow penetration of DMSO. The cells are then cooled at a rate of −1 °C/min in a standard freezing container down to −80 °C before moving the frozen cell suspension to liquid nitrogen storage (−196 °C) [50]. A non-toxic alternative or addition to DMSO would be beneficial to increase cryopreservation efficacy and potentially reduce the amount of DMSO required, thereby reducing the toxic effects. Here, ApAFP752 demonstrated significant extra- and intracellular cryoprotective activity. By transfecting AFP into cells, the AFP is given the ability to protect cells from within, improving its cryoprotective potency compared to when it is confined to the extracellular medium. This transient transfection also allows for AFP expression and cryoprotection in a non-heritable manner.

The trypan blue assay showed a significant increase in post-thaw viability for HEK 293T cells transfected with EGFP–ApAFP752 (IC AFP) (Figure 2). Interestingly, though viability was optimized at 10% DMSO, continued cryoprotection and potential attenuation of cytotoxic levels of DMSO was observed for IC AFP at 15 and 20% DMSO (Figure 2 and Figure 3) [56]. Though extracellular TrxA-ApAFP752 (EC AFP) also provided a significant increase in post-thaw viability, IC AFP demonstrated significantly higher post-thaw viability for the 0% and 5% DMSO concentrations (Figure 5A,B), and at 10% DMSO, 15 µM EC AFP displayed the same increased viability as IC AFP, with no significant difference between the two conditions (Figure 5C). This could be due to the fact that the post-thaw viability with 10% DMSO alone was an average of 66% (Table S1), and there was less room for improvement. Interestingly, there was significantly improved post-thaw viability for the combined approach of 15 µM EC and IC AFP over IC AFP alone at 0 and 10% DMSO, while at 5% DMSO, there was no statistically significant change in viability (Figure 5 and Table S1). Overall, 15 µM EC and IC AFP yielded the most potent cryoprotective activity across every DMSO concentration tested. For example, at 10% DMSO, 15 µM EC and IC AFP HEK 293T cells demonstrated 92% viability on average after freeze/thaw (Figure 5 and Table S1). It is also worth noting at 5% DMSO, IC AFP offers improved cryoprotective activity over 10% DMSO alone (Figure 2 and Figure 5, Table 1 and Table S1), demonstrating IC AFP can be implemented as a means of reducing DMSO. Additionally, 5% DMSO with 5 and 15 µM EC and IC AFP gives similar post-thaw viability as 10% DMSO with 5 and 15 µM EC AFP (Figure 5 and Table S1). This further demonstrates that EC and IC AFP can be used to reduce the amount DMSO required for cryoprotection.

The LDH release assay results were in agreement with the trypan blue viability results, only with the LDH release assay, cell damage is quantified. IC AFP significantly reduced the amount of LDH released by cells at 0, 5, 10, and 15% DMSO concentrations (Figure 3 and Figure 5, Table 2, Table 3 and Table S2). No significant decreases in LDH release were detected for the combined approach of 5 and 15 µM EC and IC AFP over IC AFP alone, indicating that IC AFP is mainly responsible for the significant decrease in LDH release (Figure 5D–F, Table 4 and Table S2). For the MTS assay, no significant differences in the relative metabolic activity measurements across treatments for each DMSO concentration demonstrated the accuracy of these initial viability counts (Figure 4 and Figure 6). It should be noted that cells exhibiting low metabolic activity, as at confluence, are still alive [57,58,59]. The consistent reduction in post-thaw metabolic activity was not surprising, as cryopreserved mammalian cells have been shown to have reduced cellular proliferation as measured by metabolic activity, and cells can take up to 96 h to recover pre-freeze proliferation rates [60,61,62].

A major role of AFPs is the inhibition of ice recrystallization during the thawing process and in the frozen state during temperature cycling. Ice recrystallization refers to the phenomenon that larger ice crystals grow more preferentially than smaller ones in order to minimize the total surface energy. The small ice crystals fuse together, increase their size significantly, and cause physical damage to cells. Inhibition of ice recrystallization is important in the control of crystal size in cryopreservation of cells and tissues [63,64,65]. Another distinction between AFPs relates to their measured ability to create a gap between the freezing point and melting point of water, known as thermal hysteresis activity (THA), and in this regard, AFPs are considered either moderately active or hyperactive [66,67]. Possibly more important for cryopreservation is that moderately active and hyperactive AFPs also differ in their ice crystal-shaping ability. Moderately active AFPs bind to the prism and/or pyramidal planes of ice, limiting ice crystal expansion to the c-axis, resulting in a needle-like ice crystal shape [20,67,68]. Hyperactive AFPs bind to both the basal and prism planes of ice, restricting ice crystal growth along all axes, and resulting in a rounded ice crystal shape [20,69]. The fusion TrxA-ApAFP752 antifreeze activity has been previously characterized by us and others for ice-recrystallization inhibition activity and affecting ice crystal size and shape [36,40,41] and THA [36,41]. We have shown that purified Trx-ApAFP752 demonstrates functional ice-recrystallization inhibition behavior at 5 μM or higher concentrations [41], which is why we used 5 and 15 μM concentrations for the extracellular AFP assays. Ice-recrystallization inhibition activity, and ice shaping are likely much more important contributors to cryoprotection in cells than THA, which does not likely play much of a role when cells are stored at cryogenic temperatures.

Previous studies using hyperactive AFPs as part of the extracellular freezing solution have shown increased cryoprotective activity in mammalian cells [33,40]. The aforementioned differences between hyperactive and moderately active AFPs may explain why improved intracellular cryoprotective activity was observed here compared to previous studies using moderately active AFPs [25]. It should be noted that these previous studies used different mammalian cells, and our findings with HEK 293T cells may not apply to all mammalian cells. The methodology used here was also vastly different than any previous study. By transfecting mammalian cells and expressing AFP within them, there are minimal manipulations necessary to obtain intracellular AFP. This ensures cells are not exposed to any additional stresses prior to freezing. This approach yielded stronger cryoprotection by ApAFP752 than our previous studies in which we either supplemented the freezing medium for human skin fibroblast cells with purified TrxA-ApAFP752 [40] or microinjected amphibian cells (frog *Xenopus laevis* eggs and embryos) with the protein [41].

GFP-AFP fusion proteins have been shown to retain or even enhance the cryoprotective activity of similar insect AFPs such as RiAFP and TmAFP [31,67]. This is thought to be due to the increased size of the fusion protein from the 27 kDa GFP combined with the 12.8 kDa (RiAFP) or 9 kDa (TmAFP) AFPs [70,71,72]. These data are further corroborated by our findings here. By transfecting AFP into cells, the otherwise non-penetrating cryoprotectant AFP is given the ability to protect the cells from within, as penetrating cryoprotectants do. This intracellular cryoprotective activity is increased for AFP in a manner similar to studies of intracellular delivery of other non-penetrating CPAs [52]. Including extracellular AFP along with intracellular AFP may provide the best cryoprotection.

One of the main goals of this study was to use well-established technologies and methods and to maintain a straightforward experimental design that builds off of current protocols. In addition, the tests performed are rapid, commercially available, and relatively low cost, making them suitable options for virtually all cell culture facilities [12]. Furthermore, these protocols follow best practices and combine several types of assays (membrane integrity, metabolic activity, etc.) necessary to achieve a comprehensive assessment of cryopreservation efficacy [73]. Because cell viability after freeze/thaw is increased with intracellular AFP, other types of cells may be studied and a unique, cryoprotected cell line with stable and inheritable AFP expression can be developed with numerous, far-reaching practical applications. This work sheds light on potential improvements to current cryopreservation protocols by the addition of an AFP transfection step 48 h prior to freezing, while still using widely available and well-established methods. For example, cells especially susceptible to cryoinjury, such as immune cells, could be transiently transfected with AFP prior to freezing to improve post-thaw cell viability [74,75]. Within just a few replication cycles after thawing, these cells would no longer produce AFP and could be used for other research purposes, including plasmid transfection and drug testing. Further studies could also include determining localization of EGFP–AFP in mammalian cells to help elucidate any membrane localization during freezing using new methods of confocal microscopy on frozen samples [76].

## 5. Conclusions

This study presented insight into differences between the extracellular and intracellular cryoprotective activity of AFP. This proof of concept shows a means of effective intracellular delivery of AFP yielding both biologically and statistically significant increases in cell viability following cryopreservation compared to current protocols. Reducing or ultimately eliminating DMSO is the ultimate goal for expanding and improving cellular storage, vaccine, and therapeutic methods seeking to avoid its toxic effects, but still retain effective means of cryopreservation.

## Figures and Tables

**Table 1:** Average % viability of HEK 293T cells across treatments as determined by trypan blue assay.

	0% DMSO	5% DMSO	10% DMSO	15% DMSO	20% DMSO
Untransfected	5	46	72	60	37
EGFP	10	42	70	62	44
IC AFP	31	80	89	77	62
IC AFP vs. Untransfected	+26 (***)	+34 (***)	+17 (***)	+17 (***)	+25 (***)
IC AFP vs. EGFP	+21 (***)	+38 (***)	+19 (***)	+15 (***)	+18 (***)

All experiments contained 3 biological repeats (*n* = 3), with each containing 3 technical repeats. *** *p* ≤ 0.001.

**Table 2:** Average LDH release of HEK 293T cells across treatments expressed as % total cellular LDH.

	0% DMSO	5% DMSO	10% DMSO	15% DMSO	20% DMSO
Untransfected	80	47	29	42	46
EGFP	81	45	30	40	42
IC AFP	66	25	16	25	32
IC AFP vs. Untransfected	−14 (*)	−22 (***)	−13 (*)	−17 (**)	−14 (ns)
IC AFP vs. EGFP	−15 (*)	−20 (***)	−14 (*)	−15 (**)	−10 (ns)

All experiments contained 3 biological repeats (*n* = 3), with each containing 3 technical repeats. n.s. *p* > 0.05, * *p* ≤ 0.05, ** *p* ≤ 0.01, *** *p* ≤ 0.001.

**Table 3:** Average increased % viability of HEK 293T cells across treatments for extracellular (EC) AFP and intracellular (IC) AFP vs. cells without AFP (untransfected), as determined by trypan blue assay.

	5 µM EC AFP	15 µM EC AFP	IC AFP	5 µM EC and IC AFP	15 µM EC and IC AFP
0% DMSO	+12 (***)	+16 (***)	+24 (***)	+24 (***)	+32 (***)
5% DMSO	+19 (***)	+18 (***)	+27 (***)	+29 (***)	+34 (***)
10% DMSO	+14 (***)	+20 (***)	+20 (***)	+23 (***)	+26 (***)

All experiments contained 3 biological repeats (*n* = 3), with each containing 3 technical repeats. *** *p* ≤ 0.001.

**Table 4:** Average LDH release of HEK 293T cells expressed as % total cellular LDH across treatments for extracellular (EC) AFP and intracellular (IC) AFP vs. cells without AFP.

	5 µM EC AFP	15 µM EC AFP	IC AFP	5 µM EC and IC AFP	15 µM EC and IC AFP
0% DMSO	−15 (ns)	−13 (ns)	−23 (*)	−22 (*)	−24 (**)
5% DMSO	−17 (*)	−15 (*)	−31 (***)	−32 (***)	−34 (***)
10% DMSO	−14 (*)	−13 (ns)	−20 (***)	−19 (***)	−20 (***)

All experiments contained 3 biological repeats (*n* = 3), with each containing 3 technical repeats. n.s. *p* > 0.05, * *p* ≤ 0.05, ** *p* ≤ 0.01, *** *p* ≤ 0.001.

## Data Availability

All data reported are included and represented in the manuscript.

## Supplementary Materials

The following supporting information can be downloaded at: https://www.mdpi.com/article/10.3390/biom12050669/s1, Figure S1: Plasmid map of EGFP–ApAFP752; Figure S2: Gating strategy for flow cytometry assessment of EGFP–AFP transfection; Figure S3: The 24 and 48 h post-transfection microscopy images; Table S1: Average increased % viability of HEK 293T cells across treatments for extracellular (EC) AFP and intracellular (IC) AFP and comparisons vs. cells without AFP (untransfected), as determined by trypan blue assay; Table S2: Average LDH release of HEK 293T cells expressed as % total cellular LDH across treatments for extracellular (EC) AFP and intracellular (IC) AFP and comparisons vs. cells without AFP (untransfected).
