# Supplementary material for: Intracellular and Extracellular Antifreeze Protein Significantly Improves Mammalian Cell Cryopreservation
Source: Biomolecules. 2022 May 5;12(5):669. doi: 10.3390/biom12050669 (PMC9139014; doi:10.3390/biom12050669)
Supplement: Supplementary file 1 [file biomolecules-12-00669-s001.zip › biomolecules-1631645-supp proof done.pdf]

Supplementary Information

Intracellular and Extracellular Antifreeze Protein Significantly Improves Mammalian Cell Cryopreservation

Jonathan A. Sreter [1], Thomas L. Foxall [2], and Krisztina Varga [1,*]

[1] Department of Molecular, Cellular and Biomedical Sciences, University of New Hampshire, Durham, NH, 03824, USA
[2] Department of Biological Sciences, University of New Hampshire, Durham, NH, 03824, USA
* Correspondence: krisztina.varga@unh.edu; Tel.: (603) 862-5375

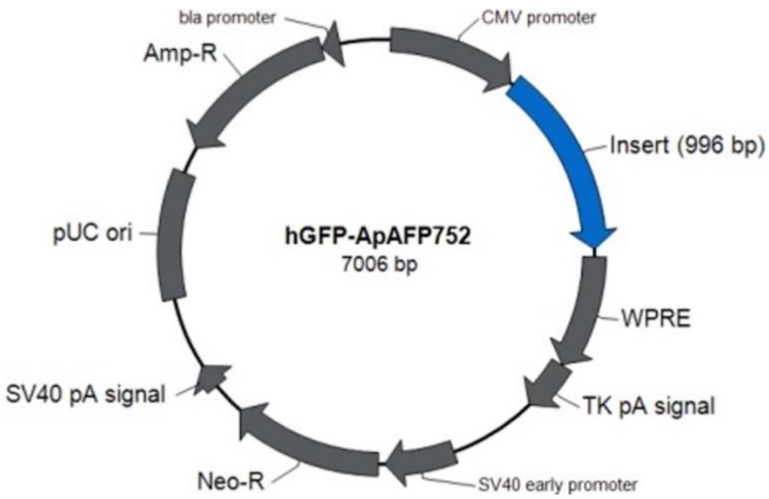

Figure S1. Plasmid map of EGFP-ApAFP752.

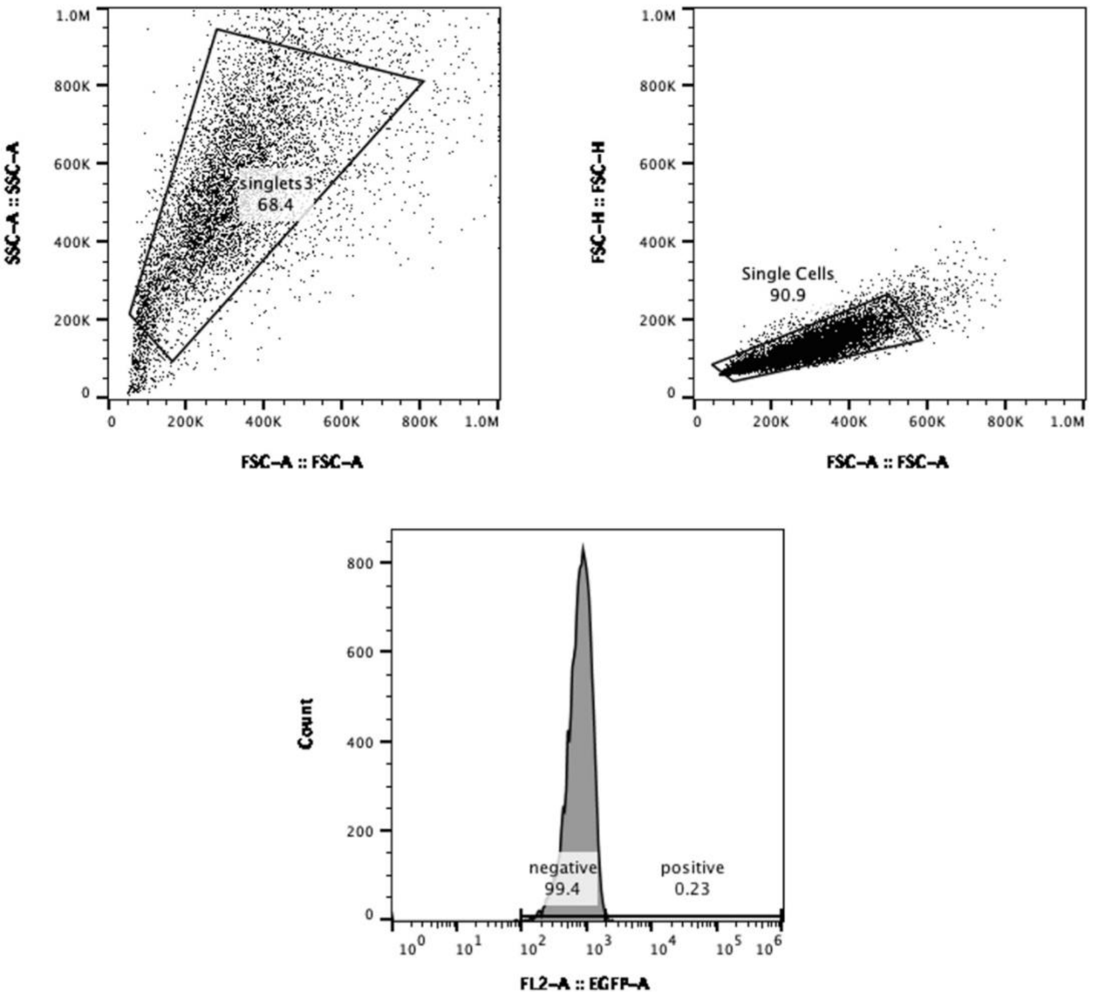

Figure S2. Gating strategy for flow cytometry assessment of EGFP-AFP transfection.

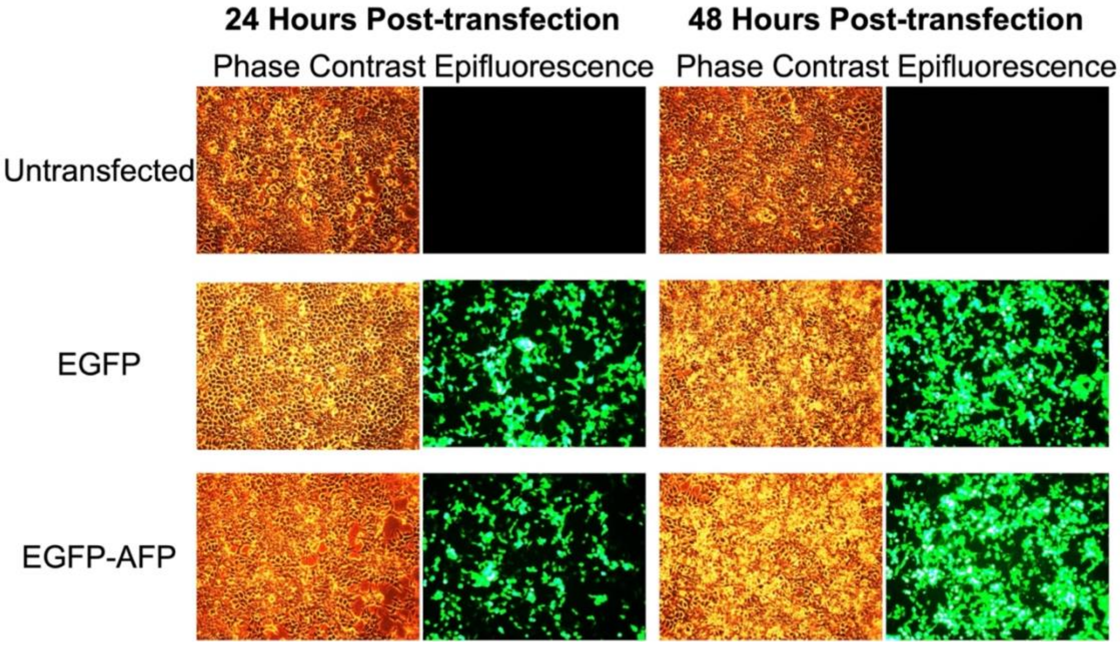

Figure S3: 24- and 48-hour post-transfection microscopy images. All images were photographed at 100X.

Table S1. Average increased % viability of HEK 293T cells across treatments for extracellular (EC) AFP and intracellular (IC) AFP and comparisons vs. cells without AFP (untransfected), as determined by trypan blue assay.

	**0% DMSO**	**5% DMSO**	**10% DMSO**
**Untransfected**	7	51	66
**5 µM EC AFP**	19	70	80
**15 µM EC AFP**	23	69	86
**IC AFP**	31	78	86
**5 µM EC & IC AFP**	31	80	89
**15 µM EC & IC AFP**	39	85	92
**5 µM EC AFP vs. Untransfected**	12 (***)	+19 (***)	+14 (***)
**15 µM EC AFP vs. Untransfected**	+16 (***)	+18 (***)	+20 (***)
**IC AFP vs. Untransfected**	+24 (***)	+27 (***)	+20 (***)
**5 µM EC & IC AFP vs. Untransfected**	+24 (***)	+29 (***)	+23 (***)
**15 µM EC & IC AFP vs. Untransfected**	+32 (***)	+34 (***)	+26 (***)

n = 3, ***$p \leq 0.001$

Table S2. Average LDH release of HEK 293T cells expressed as % total cellular LDH across treatments for extracellular (EC) AFP and intracellular (IC) AFP and comparisons vs. cells without AFP (untransfected).

	0% DMSO	5% DMSO	10% DMSO
**Untransfected**	84	54	35
**5 μM EC AFP**	69	37	21
**15 μM EC AFP**	71	39	22
**IC AFP**	61	23	15
**5 μM EC & IC AFP**	62	22	16
**15 μM EC & IC AFP**	60	20	15
**5 μM EC AFP vs. Untransfected**	-15 (ns)	-17 (*)	-14 (*)
**15 μM EC AFP vs. Untransfected**	-13 (ns)	-15 (*)	-13 (ns)
**IC AFP vs. Untransfected**	-23 (*)	-31 (***)	-20 (***)
**5 μM EC & IC AFP vs. Untransfected**	-22 (*)	-32 (***)	-19 (***)
**15 μM EC & IC AFP vs. Untransfected**	-24 (**)	-34 (***)	-20 (***)

n = 3, n.s. $p > 0.05$, *$p \leq 0.05$, **$p \leq 0.01$, ***$p \leq 0.001$
